# Bone SPECT-based segmented attenuation correction for quantitative analysis of bone metastasis (B-SAC): comparison with CT-based attenuation correction

**DOI:** 10.1186/s13550-019-0501-1

**Published:** 2019-03-19

**Authors:** Tadaki Nakahara, Yoshiki Owaki, Tsubasa Shindou, Kiyotaka Nakajima, Masahiro Jinzaki

**Affiliations:** Department of Radiology, Keio University School of Medicine, 35 Shinanomachi, Shinjuku-ku, Tokyo 160-8582 Japan

**Keywords:** Bone SPECT, Segmented attenuation correction, SUV, SPECT/CT, SPECT, Quantitation, Bone, Segmentation

## Abstract

**Background:**

Evidence has shown the clinical usefulness of measuring the metastatic tumor burden of bone for prognostic assessment especially in prostate cancer; quantitative evaluation by dedicated SPECT is difficult due to the lack of attenuation correction (AC) method.

We developed a novel method for attenuation correction using bone SPECT emission data (bone SPECT-based segmented attenuation correction; B-SAC) where emission data were virtually segmented into three tissues (i.e., bone, soft tissue, and air). Then, the pixel values in SPECT were replaced by 50 for the virtual soft tissue, and − 1000 for the virtual air. The replaced pixel values for the virtual bone were based on the averaged CT values of the normal vertebrae (B-SAC_N_) or the metastatic bones (B-SAC_M_). Subsequently, the processed SPECT data (i.e., SPECT value) were supposed to realize CT data (i.e., CT value) that were used for B-SAC. The standardized uptake values (SUVs) of 112 metastatic bone tumors in 15 patients with prostate cancer were compared between CTAC with scatter correction (SC) and resolution recovery (RR) and the following reconstruction conditions: B-SAC_N_ (+)SC(+)RR(+), B-SAC_M_ (+)SC(+)RR(+), uniform AC(UAC)(+)SC(+)RR(+), AC(−)SC(+)RR(+), and no correction (NC).

**Results:**

The SUVs in the five reconstruction conditions were all correlated with those in CTAC(+)SC(+)RR(+) (*p* < 0.01), and the correlations between B-SAC_N_ or B-SAC_M_ and CTAC images were excellent (*r* > 0.94). Bland-Altman analysis showed that the mean SUV differences between CTAC (+)SC(+)RR(+) and the other five reconstructions were 0.85 ± 2.25 for B-SAC_N_ (+)SC(+)RR(+), 1.61 ± 2.36 for B-SAC_M_ (+)SC(+)RR(+), 1.54 ± 3.84 for UAC(+)SC(+)RR(+), − 3.12 ± 4.97 for AC(−)SC(+)RR(+), and − 5.96 ± 4.59 for NC. Compared to CTAC(+)SC(+)RR(+), B-SAC_N_ (+)SC(+)RR(+) showed a slight but constant overestimation (approximately 17%) of the metastatic tumor burden of bone when the same threshold of metabolic tumor volume was used.

**Conclusions:**

The results of this preliminary study suggest the potential for B-SAC to improve the quantitation of bone metastases in bone SPECT when X-ray CT or transmission CT data are not available. Considering the small but unignorable differences of lesional SUVs between CTAC and B-SAC, SUVs obtained with the current version of B-SAC seem difficult to be directly compared with those obtained with CTAC.

## Background

With the development of reconstruction algorithms, quantitation in single-photon emission computed tomography (SPECT) has become feasible in clinical practice using standardized uptake value (SUV) [1, 2]. Attenuation correction (AC), scatter correction (SC), and resolution recovery (RR) are known to have important roles in quantifying tracer distribution in target organs or lesions. Among them, AC is generally performed by the use of integrated SPECT/computed tomography (CT) systems or external radioactive sources in order to estimate photon attenuation inside the scan area. Therefore, quantitative evaluation is considered practically difficult in facilities that have only dedicated SPECT equipment, although there are still many institutions implementing SPECT machines without CT portion.

Quantitation of radiotracer uptake is theoretically more reliable with SPECT than with planar scintigraphy because of the projection of several overlying structures in a planar image. However, quantitation of bone uptake is still challenging with dedicated SPECT. A lesion-to-background ratio is generally used to semi-quantify lesional radiotracer uptake on SPECT images, especially in the field of neurology, whereas a very limited number of clinical studies used the semi-quantitative index in bone SPECT [3] because of the difficulty in the standardization of region-of-interest settings. Recent SPECT/CT systems allow to calculate SUVs only with the information of patient body weight and injected dose, although the limited availability of SPECT/CT hampers the application of quantitative bone SPECT/CT to clinical multicenter studies. On the other hand, the total metastatic burden of bone on planar scintigraphy, called as bone scan index (BSI), has been established as a useful biomarker for the prediction of patient prognosis in prostate cancer [4, 5]. Therefore, we considered that it would become easier to conduct multicenter studies using quantitative SPECT data if a successful quantitation is achieved with dedicated SPECT.

The most common technique for attenuation correction without CT or external devices is the segmented attenuation correction (SAC) in which body structures are segmented into several kinds of tissues based on pixel values on SPECT [6, 7], positron emission tomography (PET) [8–10], and magnetic resonance (MR) images [11, 12]. As for SPECT, SAC has been used only for qualitative cardiac imaging, but not for oncologic imaging. In the present study, we developed a novel method for attenuation correction using bone SPECT emission data (bone SPECT-based segmented attenuation correction; B-SAC) where emission data were virtually segmented into three tissues (i.e. bone, soft tissue, and air). Considering the fact that the technique of SAC had been clinically applied to dedicated PET before the advent of PET/CT, it is worth evaluating the feasibility of B-SAC for quantitative assessment of bone SPECT compared to SPECT/CT.

Therefore, the purposes of this study were to evaluate the quantitative accuracy of B-SAC compared to other conventional reconstruction conditions and to discuss whether B-SAC can be used as an alternative to CT-based attenuation correction (CTAC).

## Methods

### Clinical bone SPECT data acquisition

Bone SPECT data in the areas (1–2 SPECT steps) covering metastatic sites of bone were obtained from 15 patients with prostate cancer 3 h after intravenous injection of 740 MBq of Tc-99m hydroxymethylene diphosphonate (Tc-99m HMDP) using a combined SPECT/CT system (Discovery NM/CT 670pro, GE Healthcare, Milwaukee, WI, USA). The acquisition protocol was as follows: 60 steps of 12 s/step, 360 degrees, a matrix of 128 × 128, low-energy high-resolution collimator, main energy window at 141 keV ± 10%, and sub energy window at 120 keV ± 5%. Low-dose X-ray CT was performed for CTAC.

### Virtual CT data preparation for bone SPECT-based segmented attenuation correction (B-SAC)

**Fig. 1:**
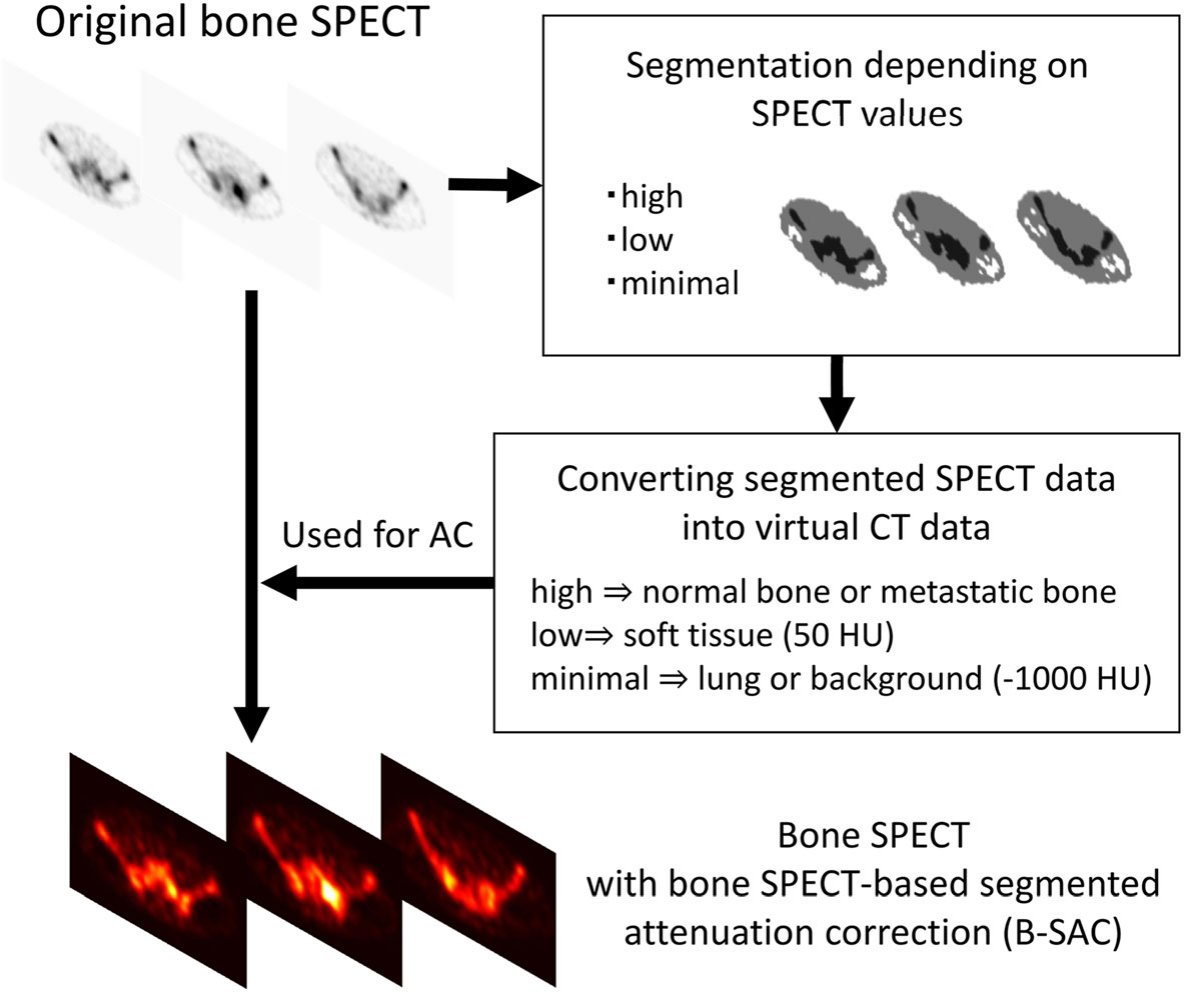
Schematic outline of the process of bone SPECT-based segmented attenuation correction (B-SAC)

**Fig. 2:**
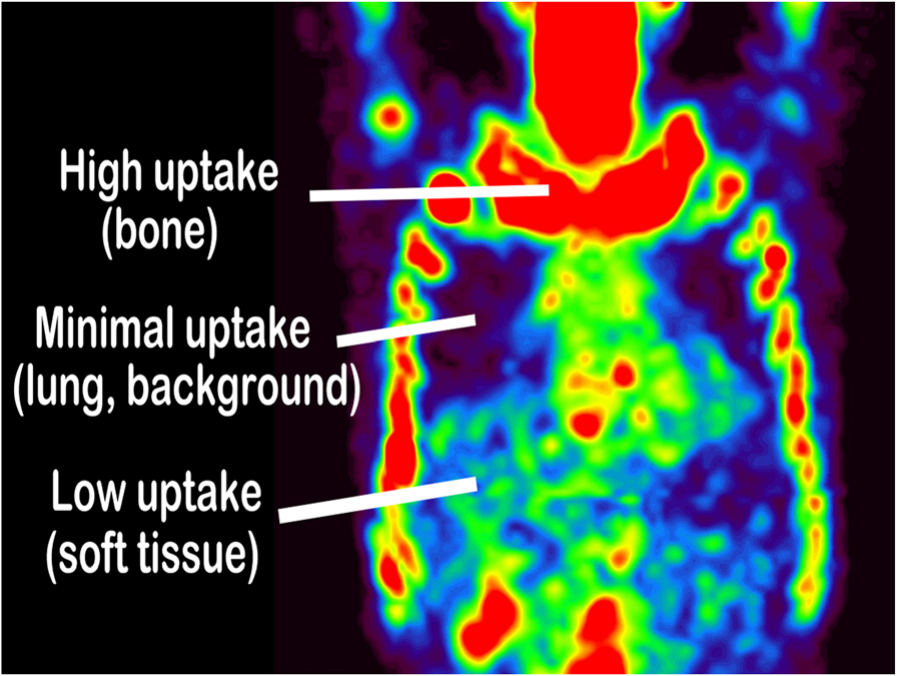
Segmentation of bone SPECT data into high, low, and minimal uptake areas for attenuation correction

The first step of generating virtual CT data for B-SAC is to segment original bone SPECT depending on SPECT pixel values (Fig. 1). The SPECT images were reconstructed according to the EANM guideline [13]: 3D-OSEM algorithm with an iteration of 5, a subset of 10, and a Gaussian filter of 4 mm. Although it is impossible to visualize the details of human anatomy from the bone SPECT images, the distribution of Tc-99m HMDP is visually divided into bone with high uptake (area A), soft tissue with low uptake (area B), and background or lung with minimal uptake (area C) (Fig. 2). In order to accomplish a semi-automatic segmentation, average lung count (L) was measured by carefully setting a spherical volume of interest (VOI) with a diameter of 60 mm in the right upper lung, and areas A, B, and C were defined as the areas of pixel values of ≥ 5 × L, pixel values of L to < 5 × L, and pixel values of < L, respectively.

Afterwards, the pixel values in bone SPECT were replaced by 50 for area B and − 1000 for area C based on general CT values for soft tissue (30–70 HU) and lung (− 900–1000 HU), respectively (Fig. 1). The replaced pixel values for area A were based on the averaged CT value of the normal vertebrae in randomly selected 40 patients without bone metastasis (B-SAC_N_) and the metastatic bones in the 15 patients with prostate cancer (B-SAC_M_), respectively. Subsequently, the processed SPECT data (i.e., SPECT value) were supposed to realize CT data (i.e., CT value) for attenuation correction. Since the virtual CT images have the same pixel size of the original SPECT images, pixel values were simply converted into attenuation coefficients without resampling.

### SPECT data reconstruction for image analysis

The clinical SPECT data were reconstructed using 3D-OSEM algorithm with an iteration of 10, a subset of 10, and a Gaussian filter of 3 mm. These reconstruction parameters were previously determined in order to perform quantitative analysis using CTAC [14]. Three kinds of attenuation correction were performed: CTAC, B-SAC, and uniform attenuation correction with Chang’s method (UAC). In UAC, 0.11 cm^−1^ was used as the attenuation coefficient according to the manufacturer’s recommendation. SC was performed using a dual-energy window method. RR by compensating the distance-dependent detector response was used. As a result, the following six types of reconstructed SPECT data were obtained: CTAC(+)SC(+)RR(+), B-SAC_N_ (+)SC(+)RR(+), B-SAC_M_ (+)SC(+)RR(+), UAC(+)SC(+)RR(+), AC(−)SC(+)RR(+), and no correction (NC).

### Quantitative analysis

In order to convert SPECT count density in the target into SUV, the calibration factor (CF) was determined using a cylindrical phantom with a diameter of 160 mm and a height of 150 mm filled with Tc-99m solution of known activity concentration (approximately 25 MBq) [14]. The phantom images with CTAC(+)SC(+)RR(+), UAC(+)SC(+)RR(+), AC(−)SC(+)RR(+), and NC were obtained for calculating CFs for the corresponding reconstruction conditions. CF for B-SAC was determined using phantom images with UAC(+)SC(+)RR(+) because the Tc-99m solution did not contain any attenuation medium.

On the phantom images, a circular region of interest was drawn on the center of the phantom in the central slice as well as in slices ± 1 and ± 2 cm away, measuring SPECT count density (count/cc). CF was calculated as the ratio of actual radioactivity concentration (as measured by the dose calibrator) in the phantom at the time of scanning to the measured SPECT count density per scan duration (Table 1).

**Table 1:** Calibration factors to convert SPECT count density into SUV

	Reconstruction conditions			
	CTAC(+)SC(+)RR(+)	UAC(+) SC(+)RR(+)	AC(−)SC(+)RR(+)	NC
Calibration factor* (Bq/cps)	4148.7	3098.1	10,299.3	32,667.6

*Based on the phantom study

SPECT count densities in a total of 112 bone metastases were measured by setting VOIs under each reconstruction condition, and SUV was calculated as:

$$ \mathrm{SUV}={\mathrm{CF}}_{\left[\mathrm{Bq}/\mathrm{cps}\right]}\times \mathrm{SPECT}\ {\mathrm{count}\ \mathrm{density}}_{\left[\mathrm{count}/\mathrm{cc}\right]}\times {\frac{1}{\mathrm{Scan}\ \mathrm{duration}}}_{\left[\sec \right]}\times \frac{{\mathrm{Body}\ \mathrm{weight}}_{\left[\mathrm{g}\right]}}{{\mathrm{Injected}\ \mathrm{activity}}_{\left[\mathrm{Bq}\right]}} $$

where SUV is defined as the ratio of the radioactivity concentration in a target to the average radioactivity concentration in the body. Then, peak SUV (SUVpeak) was measured. SUVpeak represents the average SUV obtained within a 1-cc sphere of interest centered on a highest voxel of the VOI. CTAC(+)SC(+)RR(+) image was used as a gold standard for assessing the accuracy of SUV of each metastatic lesion on the other five sets of SPECT images.

After determining which reconstruction type was the closest to CTAC(+)SC(+)RR(+), the total bone uptake (TBU; unit, gram) of bone metastases, which was calculated as the summation of metabolic tumor volume (MTV; unit, cm^3^) multiplied by its mean SUV, was compared between the reconstruction type and CTAC(+)SC(+)RR(+). Regarding the definition of MTV, 41% of SUVmax of the target was recommended for contouring tumors on PET [15], and some studies used higher thresholds (i.e., 50–60%) [16–18]. Considering the poorer spatial resolution of SPECT than that of PET, we set the VOIs delineating 50%, 55%, and 60% of SUVmax within the lesions to delineate the metastatic lesions on SPECT. Then, TBUs were measured as TBU_50_, TBU_55_, and TBU_60_.

### Statistical analysis

All statistical data analyses were performed using SPSS version 22.0 (IBM Corporation). Correlations of SUV between CTAC(+)SC(+)RR(+) image and the other five sets of images were assessed with the Pearson correlation coefficient. The Bland-Altman analysis was applied for evaluating the agreement of SUV measurement between CTAC(+)SC(+)RR(+) image and each of the five SPECT images.

## Results

### Replacing pixel values of normal and metastatic bones for B-SAC

Averaged CT values of the normal vertebrae and metastatic bones were 289.1 ± 42.4 and 631.9 ± 314.7, respectively. Therefore, the pixel values in bone SPECT for area A were replaced by 300 for B-SAC_N_ and 600 for B-SAC_M_.

### Correlations and differences between CTAC(+)SC(+)RR(+) and the other five reconstructions in the quantitative measurement of bone metastases

**Fig. 3:**
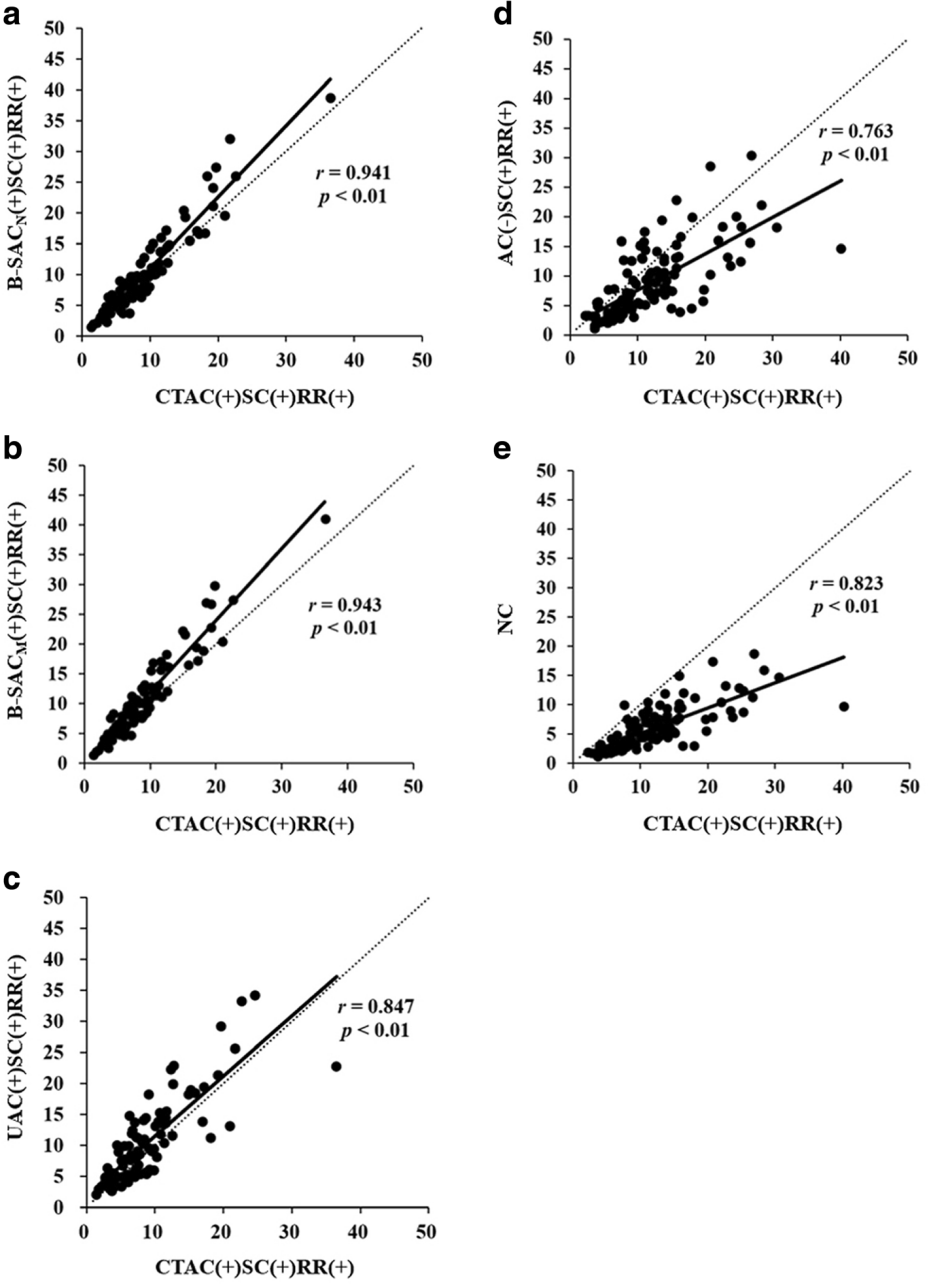
Correlations between CTAC(+)SC(+)RR(+) and the other 5 reconstructions in the SUV measurement of bone metastases. **a**: B-SAC_N_ (+)SC(+)RR(+). **b**: B-SAC_M_ (+)SC(+)RR(+). **c**: UAC(+)SC(+)RR(+). **d**: AC(-)SC(+)RR(+). **e**: NC

SUVs of the 112 metastatic bone tumors in B-SAC_N_ (+)SC(+)RR(+), B-SAC_M_ (+)SC(+)RR(+), UAC(+)SC(+)RR(+), AC(−)SC(+)RR(+), and NC images were all correlated with SUVs in CTAC(+)SC(+)RR(+) images (*p* < 0.01) (Fig. 3). Especially, the correlations of SUVs between B-SAC and CTAC images were excellent (*r* > 0.94).

**Fig. 4:**
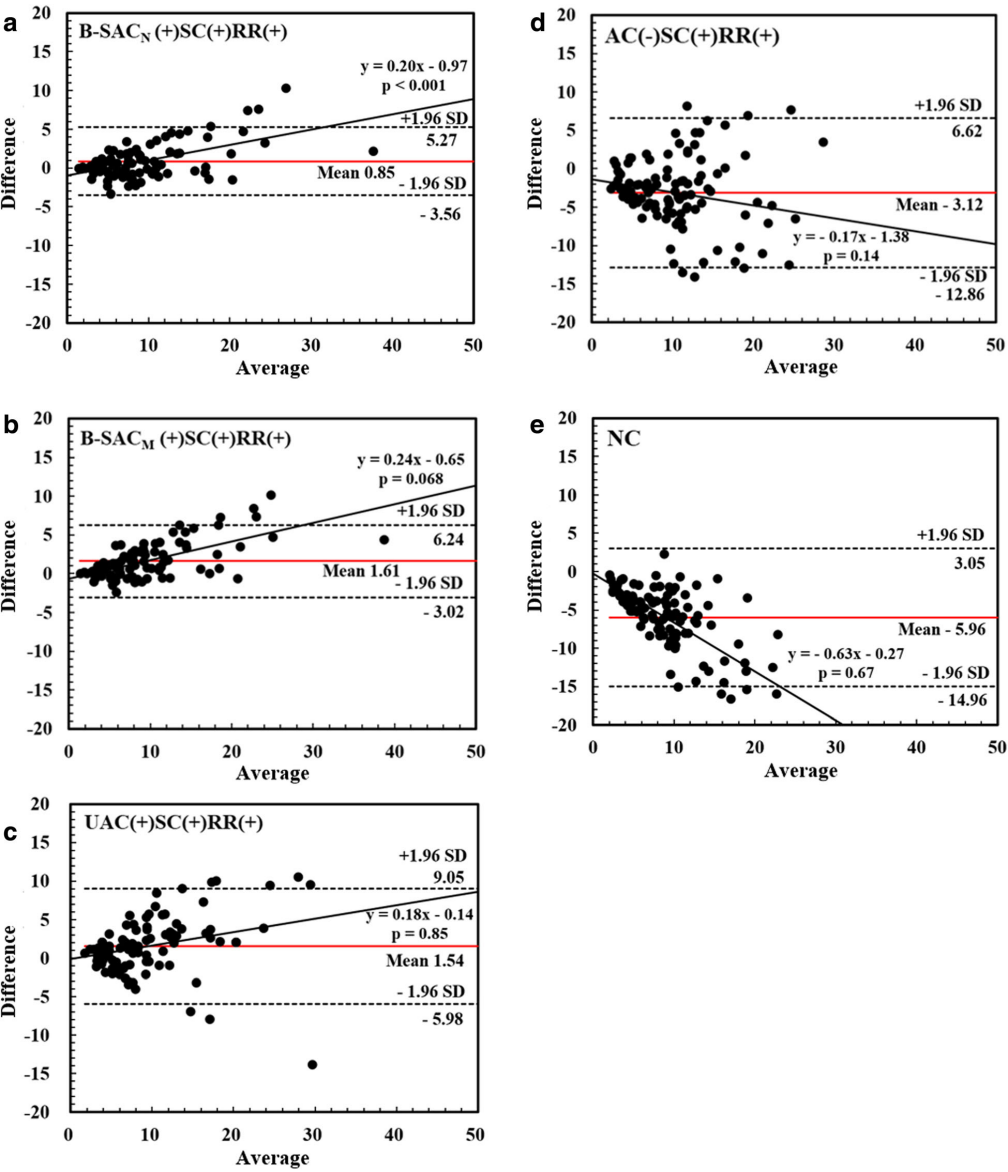
Bland-Altman plots of SUVs of bone metastases between CTAC(+)SC(+)RR(+) and the other five reconstruction conditions. **a** B-SAC_N_ (+)SC(+)RR(+). **b** B-SAC_M_ (+)SC(+)RR(+). **c** UAC(+)SC(+)RR(+). **d** AC(−)SC(+)RR(+). **e** NC

The Bland-Altman analysis (Fig. 4) showed that the mean SUV differences between CTAC(+)SC(+)RR(+) and the other five reconstructions were 0.85 ± 2.25 for B-SAC_N_ (+)SC(+)RR(+), 1.61 ± 2.36 for B-SAC_M_ (+)SC(+)RR(+), 1.54 ± 3.84 for UAC(+)SC(+)RR(+), − 3.12 ± 4.97 for AC(−)SC(+)RR(+), and − 5.96 ± 4.59 for NC. Ninety-five percent limits of agreement ranged between − 3.56 to 5.27 for B-SAC_N_ (+)SC(+)RR(+), − 3.02 to 6.24 for B-SAC_M_ (+)SC(+)RR(+), − 5.98 to 9.05 for UAC(+)SC(+)RR(+), − 12.86 to 6.62 for AC(−)SC(+)RR(+), and − 14.96 to 3.05 for NC. There was a significant proportional bias between B-SAC_N_ (+)SC(+)RR(+) and CTAC(+)SC(+)RR(+). The lesional SUVs were relatively higher with B-SAC than with CTAC (ratio, 1.08 ± 0.22). In addition, the tendency of the bias between B-SAC_M_ (+)SC(+)RR(+) and CTAC(+)SC(+)RR(+) was noted, whereas there were no proportional biases between the other three reconstructions and CTAC(+)SC(+)RR(+).

**Fig. 5:**
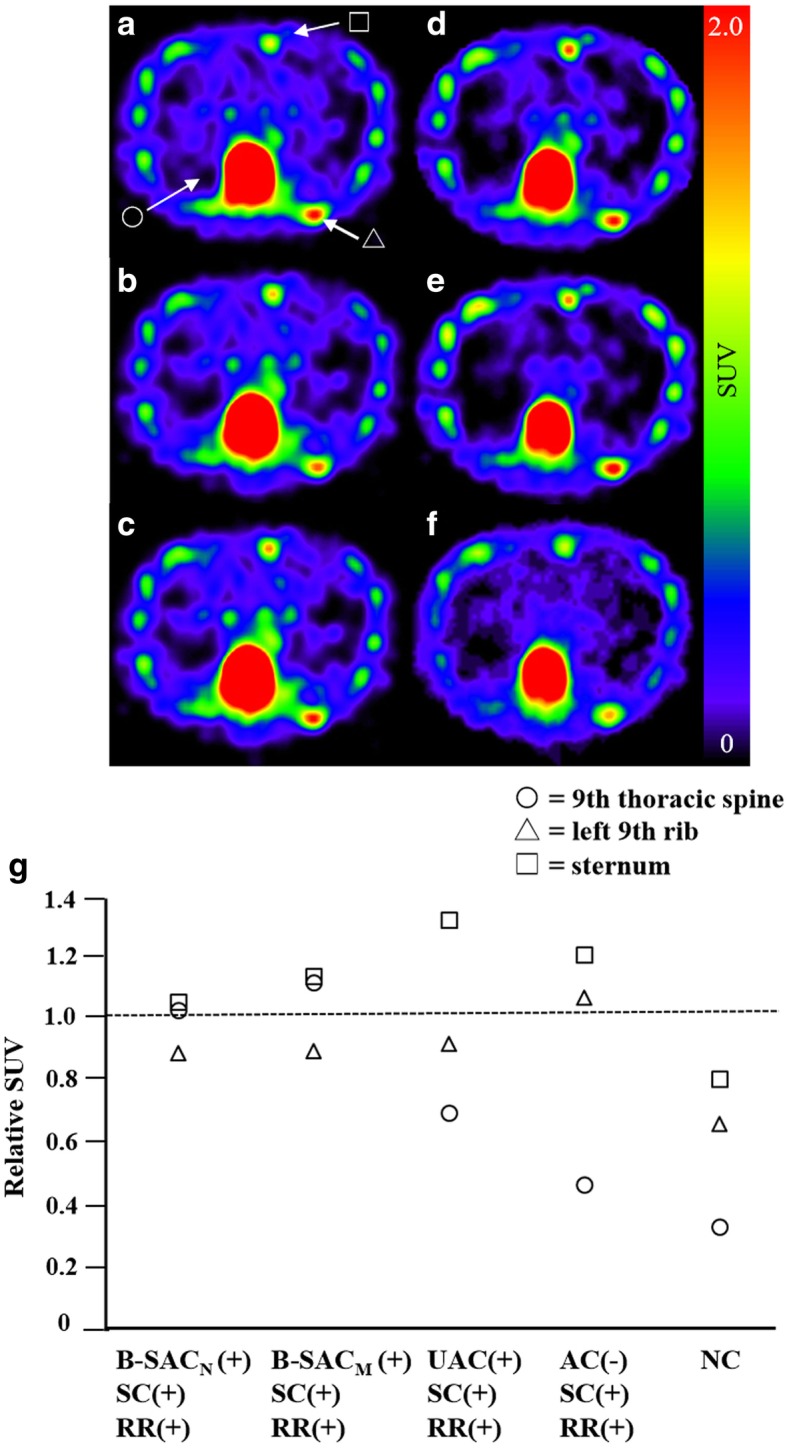
Comparison of CTAC(+)SC(+)RR(+) and the other five reconstruction conditions in SUV measurement in a patient with bone metastases in the thoracic spine, rib, and sternum. **a** CTAC(+)SC(+)RR(+). **b** B-SAC_N_ (+)SC(+)RR(+). **c** B-SAC_M_ (+)SC(+)RR(+). **d** UAC(+)SC(+)RR(+). **e** AC(−)SC(+)RR(+). **f** NC. **g** Relative SUVs observed with the reconstructions **a**–**f** to those observed with CTAC(+)SC(+)RR(+)

These results indicated B-SAC_N_(+)SC(+)RR(+) was found to be the closest to CTAC(+)SC(+)RR(+) in terms of SUV measurement of the bone metastases, although B-SAC_N_ showed a slight overestimation compared to CTAC. A representative case was shown in Fig. 5.

### Comparison of TBU between B-SAC_N_ (+)SC(+)RR(+) and CTAC(+)SC(+)RR(+)

B-SAC_N_ (+)SC(+)RR(+), which was best correlated with CTAC(+)SC(+)RR(+) regarding SUV, was further compared regarding the TBUs of bone metastases on a per-patient basis. TBUs in B-SAC_N_ (+)SC(+)RR(+) were slightly higher than those in CTAC(+)SC(+)RR(+) when the same threshold of MTV was used; the ratios of TBU_50_, TBU_55_, and TBU_60_ in the former to TBU_50_, TBU_55_, and TBU_60_ in the latter were 1.17 ± 0.22, 1.17 ± 0.22, and 1.17 ± 0.21, respectively (Table 2). These results were definitely due to the overestimation of SUV observed with B-SAC compared to CTAC. Therefore, an additional comparison was made to determine how much higher threshold should be set in B-SAC to achieve closer TBU than the predetermined threshold (i.e., 50%) in CTAC: TBU_55_ and TBU_60_ in B-SAC_N_ (+)SC(+)RR(+) vs TBU_50_ in CTAC(+)SC(+)RR(+). The ratios were 0.96 ± 0.19 and 0.77 ± 0.17 (Table 2).

**Table 2:** Differences in the total bone uptake (TBU) between B-SAC and CTAC

	TBU [g] (range)		Ratio of TBUs between B-SAC and CTAC	
Threshold for metabolic tumor volume	B-SAC	CTAC	Same threshold*	50% in CTAC**
50%	902 ± 375 (359–1617)	821 ± 434 (299–1665)	1.17 ± 0.22	1.17 ± 0.22
55%	743 ± 304 (287–1186)	676 ± 351 (242–1241)	1.17 ± 0.22	0.96 ± 0.19
60%	591 ± 241 (228–838)	537 ± 276 (189–869)	1.17 ± 0.21	0.77 ± 0.17

*Same threshold for metabolic tumor volume in both B-SAC and CTAC

**Threshold for metabolic tumor volume was set at 50% in CTAC

## Discussion

There have still been many institutions implementing only dedicated SPECT. However, its inability to perform quantitative analysis reduces the value of bone SPECT, which may account for increased high-quality evidence of bone planar scintigraphy instead of bone SPECT. Recently, quantitative bone SPECT/CT was reported to provide more accurate assessment of bone metastasis than planar scintigraphy [19], suggesting that bone SPECT with B-SAC could have better performance than planar scintigraphy when assessing the metastatic tumor burden. Furthermore, B-SAC may allow to estimate the delivered dose of therapeutic radionuclides such as Ra-223 to metastatic lesions on a per-lesion basis because of an easier evaluation of individual bone metastasis than planar scintigraphy. Therefore, an achievement of quantitation with dedicated SPECT would expand its clinical usability considering the fact that cancer is now a common disease.

### Importance of attenuation correction for quantitation

We found that SUVs in all of the five evaluated images (B-SAC_N_ (+)SC(+)RR(+), B-SAC_M_ (+)SC(+)RR(+), UAC(+)SC(+)RR(+), AC(−)SC(+)RR(+), and NC) had substantial correlations with those in CTAC(+)SC(+)RR(+) images. However, B-SAC(+)SC(+)RR(+) revealed the best correlation with CTAC(+)SC(+)RR(+), and the absolute difference between B-SAC(+)SC(+)RR(+) and CTAC(+)SC(+)RR(+) images was much smaller than those between the others and CTAC(+)SC(+)RR(+) images. Even uniform attenuation correction, which is widely applied to quantitative brain SPECT, was found to be insufficient as a method of attenuation correction for bone SPECT. These results clearly suggested that an attenuation correction of bone as well as soft tissues plays an important role in the quantitation of bone uptake.

### Segmentation according to pixel value in bone SPECT

A major advantage of using bone SPECT emission data for attenuation correction is that it is feasible to semi-automatically delineate bone structures which exhibit the highly accumulated areas on SPECT images. Photon attenuation is dependent on transmission medium (usually defined as a function of attenuation coefficient) inside the body through which photon travels into the detector. If the human body is roughly divided into three components according to photon attenuation (i.e., bone, soft tissue/water, and air), bone has the greatest impact on attenuation correction whereas the attenuation effect of air is negligible. Although the other tissues than bone or air have different attenuation coefficient values (in this study the coefficient for them was constant), the difference would be much smaller than the difference between bone and soft tissue/water, or than the difference between soft tissue/water and air. Based on this assumption, the fixed values replacing areas A (300 HU or 600 HU) and B (50 HU) for B-SAC were applied to every case in spite of the non-uniform human structure. In addition, metastatic bones had different CT values. Therefore, SUVs obtained with the current version of B-SAC seem difficult to be directly compared with those obtained with CTAC.

In the present study, the cutoff values for the segmentation on original SPECT images were based on the lung counts because of the following reasons. First, the contour of the lung is roughly identified on SPECT images because of its very low background uptake and surrounding uptake in the rib, liver, and mediastinum (Fig. 2). Second, SPECT count density in the lung seems constant depending on the VOI position within the lung (e.g., peripheral or deep side of the lung) even on non-attenuation-corrected SPECT images because of minimal attenuation effect within the lung. However, it remains uncertain whether the proposed method is most suitable for B-SAC. In addition, the threshold settings for discriminating areas A, B, and C were only based on our visual inspection of the bone SPECT images in the 55 patients with prostate cancer with (*n* = 15) and without (*n* = 40) bone metastasis, which is one of the weaknesses of this study.

In B-SAC, the urinary system, known as high uptake in bone SPECT, is recognized as one of the bone tissues. Therefore, the kidney, urinary tract, and bladder show as high SPECT values as the bone even after B-SAC. However, as far as we evaluated the cases in the present study, we have observed no measurement error due to high accumulation in non-bone tissues including the urinary system. However, it is possible to cause the error especially when extraosseous accumulation is very close to any bone structure.

Moreover, the tissue segmentation with the current B-SAC method is susceptible to image noise because of the lack of the contouring process. Therefore, image smoothing and/or longer scan duration seems important when performing the segmentation. However, considerable image filtering can lower SPECT values of both normal and metastatic bones, resulting in the potential failure of bone segmentation. It is therefore necessary to perform further research about the optimization of smoothing and/or scan duration to improve the accuracy of the B-SAC.

Despite these limitations, the results of this preliminary study suggest the potential for B-SAC to improve the quantitation of bone metastases in bone SPECT when X-ray CT or transmission CT data are not available.

### Possible advantage of B-SAC over CTAC

B-SAC may contribute to the reduction of radiation dose compared to CTAC if quantitative bone uptake is a key to clinical assessments including differentiation of benign and malignant bone lesions, evaluation of active inflammatory bone diseases, and treatment evaluation of a variety of bone diseases. In pediatric patients, sports injuries, scoliosis, trauma, and bone tumors causing back pain are often evaluated with bone SPECT/CT or fluoride-18 PET/CT [20], although the radiation exposure from low-dose CT would be a matter of debate [21]. Indeed, MR-based segmented attenuation correction to pediatric PET/MR imaging has been specifically developed for generating attenuation maps without CT [11].

### TBU measurement of bone metastasis for assessing patient prognosis

Recently, a randomized controlled clinical trial succeeded in demonstrating that BSI in planar scintigraphy was a good prognostic indicator in patients with prostate cancer [5]. Another large-scale clinical trial also showed that the volume of bone metastases affected the benefit of radiotherapy to the primary prostate cancer [22]. Thus, the measurement of metastatic tumor burden is crucial for the management of prostate cancer patients, and BSI has been used as one of the biomarkers for this purpose. However, since the automatic extraction of metastatic lesions for calculating BSI is based on the image contrast between normal and metastatic lesions, it can cause an underestimation of metastatic tumor burden especially in case of diffuse bone metastases as a result of the reduced ability to differentiate between normal and pathologic uptake areas from anteroposterior planar images [19]. On the other hand, TBU is a promising alternative to BSI because of both the improved image contrast by SPECT and the objective assessment of measuring lesional SUVs. We found that TBUs observed with B-SAC were slightly and constantly (17%) higher than those observed with CTAC when the same threshold was used. These results suggested that B-SAC is probably comparable to CTAC when comparing TBUs before and after treatment. It may further facilitate the comparison of SUVs among different dedicated SPECT scanners or between dedicated SPECT and SPECT/CT if the reconstruction condition and the threshold for tumor delineation are properly adjusted. However, at present, an appropriate application of B-SAC requires full validation through significant clinical data from many institutions.

## Conclusions

We developed a novel method of attenuation correction using original bone SPECT data that we call B-SAC. SUVs in B-SAC(+)SC(+)RR(+) were best correlated with those in CTAC(+)SC(+)RR(+) compared to any other reconstruction conditions. Especially, B-SAC by replacing pixel values for bone based on the averaged CT value of the normal vertebrae (B-SAC_N_) showed an excellent correlation and agreement with CTAC. However, a certain error is unavoidable when quantifying radiotracer uptake in an individual metastatic lesion considering the process of B-SAC. Nevertheless, this study demonstrated that B-SAC may be an option for attenuation correction if quantitative analysis is required even with dedicated SPECT.

## Abbreviations

AC: Attenuation correction
B-SAC: Bone SPECT-based segmented attenuation correction
BSI: Bone scan index
CF: Calibration factor
CT: Computed tomography
CTAC: CT-based attenuation correction
HMDP: Hydroxymethylene diphosphonate
MR: Magnetic resonance
MTV: Metabolic tumor volume
PET: Positron emission tomography
RR: Resolution recovery
SAC: Segmented attenuation correction
SC: Scatter correction
SPECT: Single-photon emission computed tomography
SUV: Standardized uptake value
TBU: Total bone uptake
VOI: Volume of interest
